# Electrochemical determination of ascorbic acid using palladium supported on N-doped graphene quantum dot modified electrode

**DOI:** 10.1038/s41598-024-56231-x

**Published:** 2024-03-12

**Authors:** K. Mohammadnezhad, F. Ahour, S. Keshipour

**Affiliations:** 1 Nanotechnology Research Group, Faculty of Chemistry, Urmia University, Urmia, Iran; 2 Department of Nanochemistry, Nanotechnology Research Center, Urmia University, Urmia, Iran; 3 Central Laboratory of Urmia University, Urmia University, Urmia, Iran

**Keywords:** Electrochemical sensor, Doped graphene quantum dot, Ascorbic acid, Pd nanoparticles, Voltammetry, Electrocatalyst, Chemistry, Nanoscience and technology

## Abstract

To precise screening concentration of ascorbic acid (AA), a novel electrochemical sensor was prepared using palladium nanoparticles decorated on nitrogen-doped graphene quantum dot modified glassy carbon electrode (PdNPs@N-GQD/GCE). For this purpose, nitrogen doped GQD nanoparticles (N-GQD) were synthesized from a citric acid condensation reaction in the presence of ethylenediamine and subsequently modified by palladium nanoparticles (PdNPs). The electrochemical behavior of AA was investigated, in which the oxidation peak appeared at 0 V related to the AA oxidation. Considering the synergistic effect of Pd nanoparticles as an active electrocatalyst, and N-GQD as an electron transfer accelerator and electrocatalytic activity improving agent, PdNPs@N-GQD hybrid materials showed excellent activity in the direct oxidation of AA. In the optimal conditions, the voltammetric response was linear in the range from 30 to 700 nM and the detection limit was calculated to be 23 nM. The validity and the efficiency of the proposed sensor were successfully tested and confirmed by measuring AA in real samples of chewing tablets, and fruit juice.

## Introduction

Vitamin C or ascorbic acid (AA) is a water-soluble antioxidant vitamin with a lactone-like structure. This vitamin is known for its regenerative properties which is easily oxidized to produce dihydroascorbic acid. AA is a strong anti-cancer compound that prevents all types of free radical damage^1–7^. Although various methods have been proposed for the analysis of ascorbic acid, efforts are still being made to search for simpler, faster, more sensitive, and more selective methods. In addition to traditional methods such as titration^8^, spectrophotometric methods^9–14^, chemiluminescence^15–17^, and non-spectrophotometric measurements are also available^18–20^, which require long analysis times, and are likely to degrade AA^21^. Selective electrochemical determination of AA is the main goal of the researches, and its direct oxidation on an ordinary electrode is almost difficult due to its high potential and its surface deposition by oxidation products^21–25^. Many of the previous works paid attention to chemically modified electrodes, where the bound species are usually electrochemically active molecules^22^. When such modified electrodes are used as sensors in electroanalytical applications, they can be expected to be involved in charge transfer between the modifier on the electrode surface and the compound in the solution, leading to an increase in the response and a change in the reaction potential^26–30^.

In recent years, metallic and metal oxide nanomaterial-modified surfaces have been a matter of interest due to their electronic, catalytic, magnetic, and optical properties^31–36^. Noble metal nanoparticles (NPs) like Au, Ag, Pt, Pd, and Ru, and their alloys have emerged as a new class of compounds with broad applications and received increasing attention owing to their unique properties. Among them, Pd-based catalysts have become a hot topic of interest because of their valuable properties and have been used for making various types of electrochemical sensors for compounds having a slow redox process on the surface of bare electrodes^21^. It should be noted that NPs have different electrocatalytic activity compared to bulk materials depending on their size and morphology. The modified electrode with ultra-thin palladium nanowires has been used for the selective detection of AA^37^. PdNPs supported on graphene oxide used for the electrochemical determination of AA^38^. PdNPs-loaded on carbon nanofiber modified electrodes applied for the simultaneous electrochemical determination of dopamine (DA), uric acid (UA), and AA^39^.

GQDs have promising results as a support for metal nanoparticles and can increase the catalytic activity of supported nanoparticles by manipulating their electronic structure. It is thought that the presence of higher oxygen groups, in addition to the better dispersion of nanoparticles, leads to a relative loss of conductivity and stability of GQDs, which doping with nitrogen solves these problems. The remarkable electronegativity difference between N (3.04) and C (2.55) causes polarity in the carbon network and improves the electronic, magnetic, and optical properties of these types of materials^40,41^. Doped nitrogen results in electrocatalytic activity improvement of the metal nanoparticles by providing the necessary electron withdrawal^42^. Also, placing nitrogen in graphene-based materials increases the charge carriers and the interaction between the support and metal nanoparticles, which leads to the important role of nitrogen in electrochemical sensors^43–46^. That is, the higher electronegativity of nitrogen compared to carbon, not only does not reduce the electrical conductivity and stability of the carbon support but also provides the necessary electron withdraw to increase the activity of metal nanoparticles.

**Figure 1:**
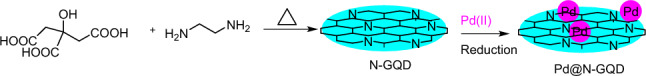
Schematic presentation of the synthesis of PdNPs@N-GQD.

Recently, our group synthesized Pd(0) supported on nitrogen-doped graphene quantum dots modified cellulose as an efficient catalyst for the green reduction of nitroaromatics with excellent properties^41^. In continuation, herein, we used PdNPs supported on N-GQD (PdNPs@N-GQD) as an electrode modifier for the preparation of sensitive and selective electrochemical sensor for AA detection. The reaction was promoted by thermal treatment of citric acid, and ethylenediamine, and subsequent deposition of PdNPs (Fig. 1).

## Experimental

### Preparation of PdNPs@N-GQD

A mixture of 4 g citric acid, and 1.5 ml ethylenediamine in 10 ml of deionized water was stirred at room temperature for 0.5 h to give a solution. Then, the obtained solution was dried at 80 °C, and heated in an autoclave at 200 °C for 2 h to give a dark solid. The obtained solid was washed with deionized water (50 ml) and dried in an oven at 60 °C^47^. N-GQD was dispersed in 10 ml of deionized water, and 0.01 g of PdCl_2_ was added to the mixture. After stirring for 0.5 h, a NaBH_4_ solution was added dropwise over 0.5 h and the solution was stirred for 2 h. Finally, the dark solid was passed through filter paper, washed out with deionized water (3 × 10 ml), and dried in an oven (60 °C).

Additional information about materials, methods, and electrochemical parameters are presented in Supplementary Information (SI).

## Results and discussion

### Characterization of PdNPs@N-GQD

**Figure 2:**
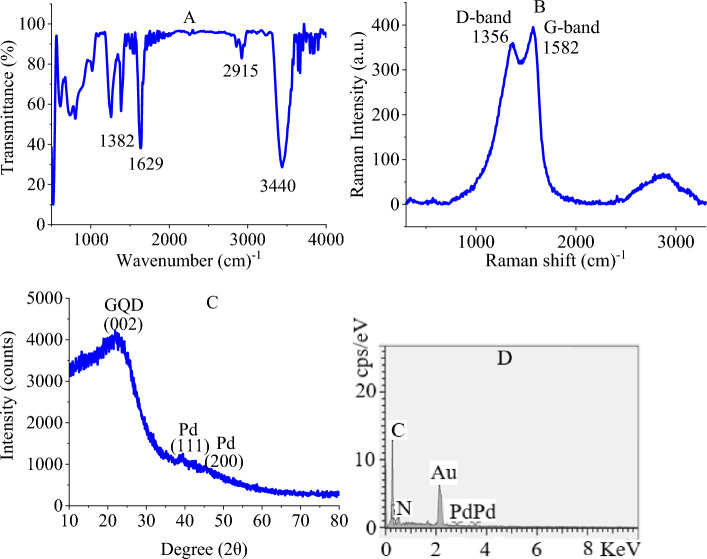
FT-IR (**A**), Raman (**B**), XRD (**C**), and EDX (**D**) of PdNPs@N-GQD.

To determine the structure of PdNPs@N-GQD, spectroscopic, and microscopic studies were conducted on the nanocomposite. Initially, the FT-IR spectrum of PdNPs@N-GQD was provided, in which the expected absorption peaks observed for stretching OH/NH at 3440 cm^−1^, stretching CH at 2915 cm^−1^, stretching C=C at 1629 cm^−1^, and bending OH at 1382 cm^−1^ (Fig. 2A)^48^.

It was crucial to approve the formation of graphene material and among various methods, Raman spectroscopy did that efficiently. The spectrum demonstrated D-band and G-band of N-GQD ascribed to out-of-plane CH, and in-plane C=C vibrations, respectively (Fig. 2B)^49^. High intensity of the D-band is a significant sign for the abundance of functional groups on the graphene sheets. Moreover, the XRD diffractogram of PdNPs@N-GQD indicated a (002) peak of N-GQD as another confirmation of the graphene nature of the support^50^. This analysis also showed (111), and (200) peaks of Pd (Fig. 2C)^51^. Atomic spectroscopy was performed to determine N and Pd. Accordingly, EDX analysis showed the presence of N, and Pd (Fig. 2D).

**Figure 3:**
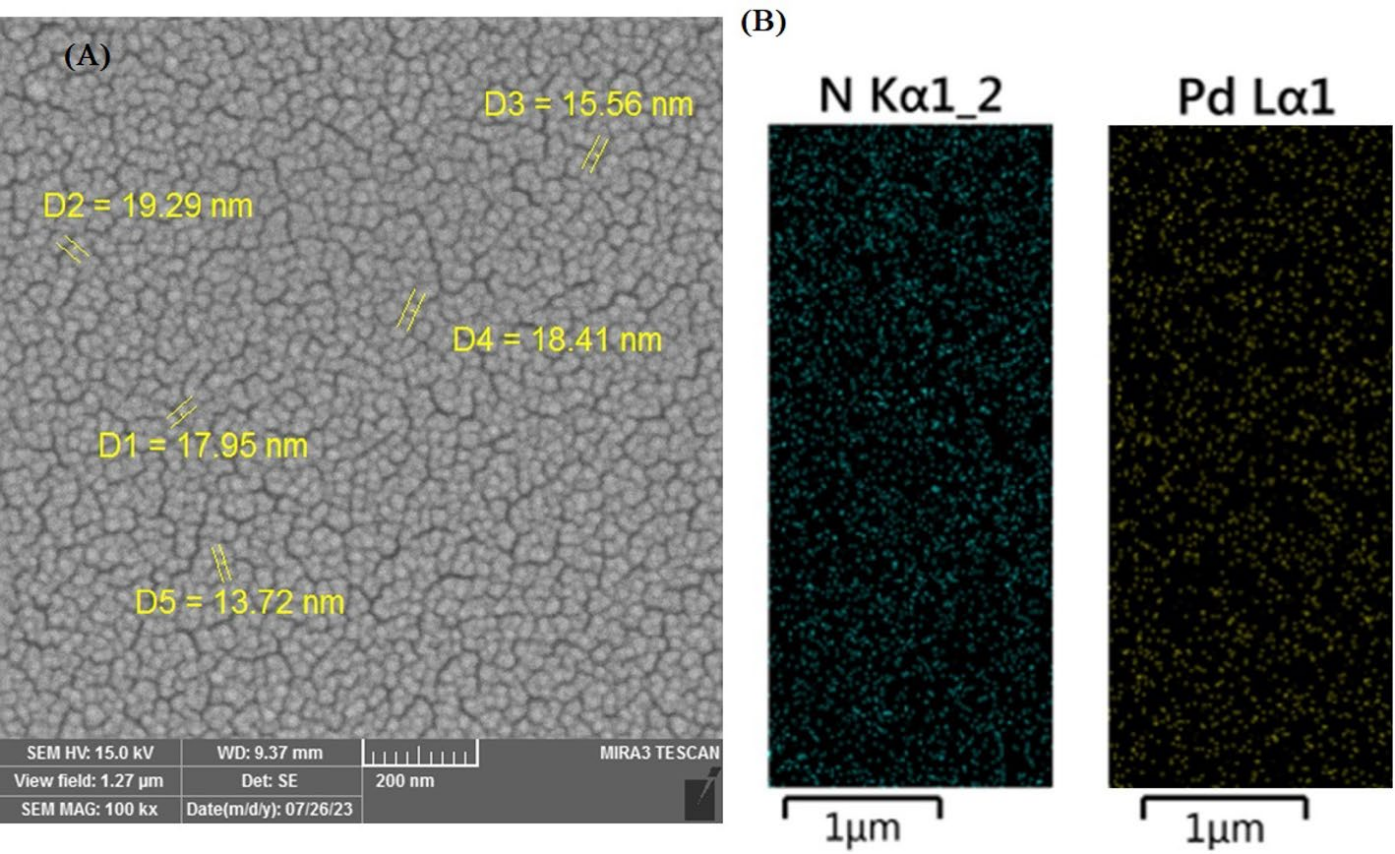
SEM (**A**), and elemental mapping (**B**) of PdNPs@N-GQD.

A micrograph of PdNPs@N-GQD was prepared to study the morphology of the nanocomposite (Fig. 3). The image indicated the formation of fine particles with a size in the range of 13–20 nm^52^. This could be attributed to the GQD since they are considered as the zero-dimension nanomaterials. The nature of nanoparticles was also studied with elemental mapping, revealing both N and Pd in the structure. The mapping showed a homogeneous distribution of Pd nanoparticles on the electrode surface, which leading to high performance (Fig. 3).

### Electrochemical evaluations of the prepared sensor

**Figure 4:**
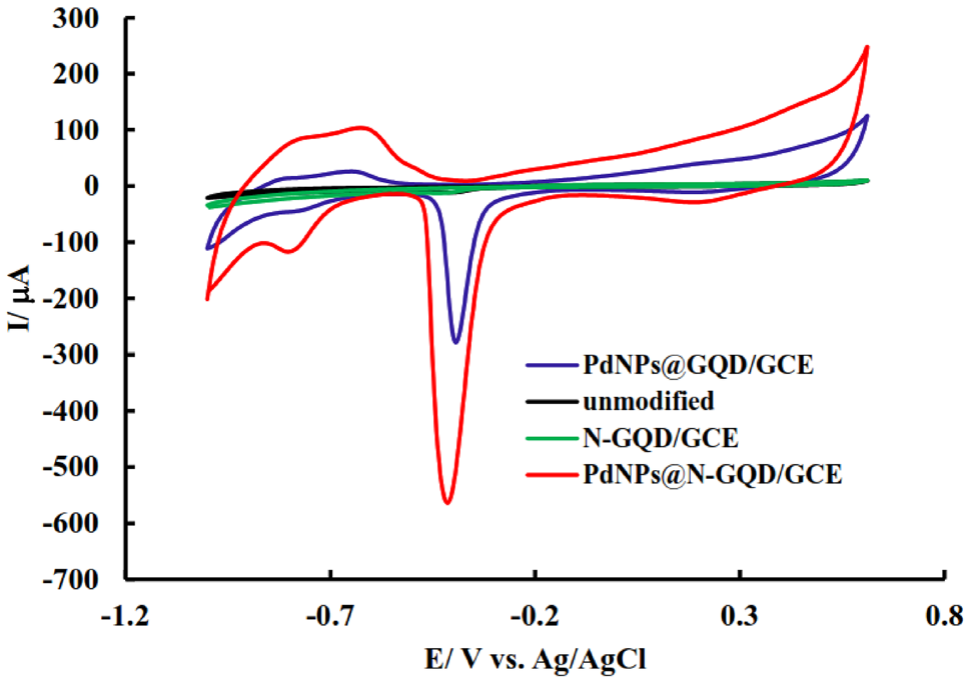
CV of unmodified, and modified electrodes (N-GQD/GCE, PdNPs@GQD/GCE, and PdNPs@N-GQD/GCE) after immersion in 0.1 M solution of NaOH; scan rate 0.1 V/s.

Firstly, to investigate the stabilization of NPs on the surface of the electrode and the effect of the modifier, the CV signal of the electrode in NaOH solution was used to confirm the stabilization of NPs at the electrode surface. For this purpose, the unmodified, and modified electrodes (N-GQD/GCE, PdNPs@GQD/GCE, and PdNPs@N-GQD/GCE) were placed inside a 0.1 M solution of NaOH, and its CV was done in the potential range 0.6 to − 1 V with a scan rate of 0.1 V/s (Fig. 4). In the resulting voltammograms, an oxidation peak appeared at a potential of 0.4 V, which according to the reports related to the electrooxidation of PdNPs. In the reverse scan, Pd oxides are reduced to Pd^0^ at − 0.4 V. This clearly shows the presence of Pd^0^ at the electrode surface and proves that the presence of N-GQD leads to a reduction in the particle size and better stabilization of PdNPs, approved by the appearance of a sharper reduction peak compared to PdNPs@GQD/GCE. Moreover, the peaks appearing at − 0.8 and − 0.6 V are related to the adsorption and desorption of hydrogen at the electrode surface due to the presence of PdNPs at the surface.

### Investigation of AA oxidation at the electrode surface

Oxidation of AA in many cases requires a high overpotential. Figure S1 shows the CV of the modified electrode in the blank electrolyte before and after the addition of AA (10 mM). The comparison of voltammograms a, and b, confirmed the oxidation of AA at a potential of approximately 0 V on the surface of the PdNPs@N-GQD/GCE.

### Investigating the electrochemical behavior of different electrodes

**Figure 5:**
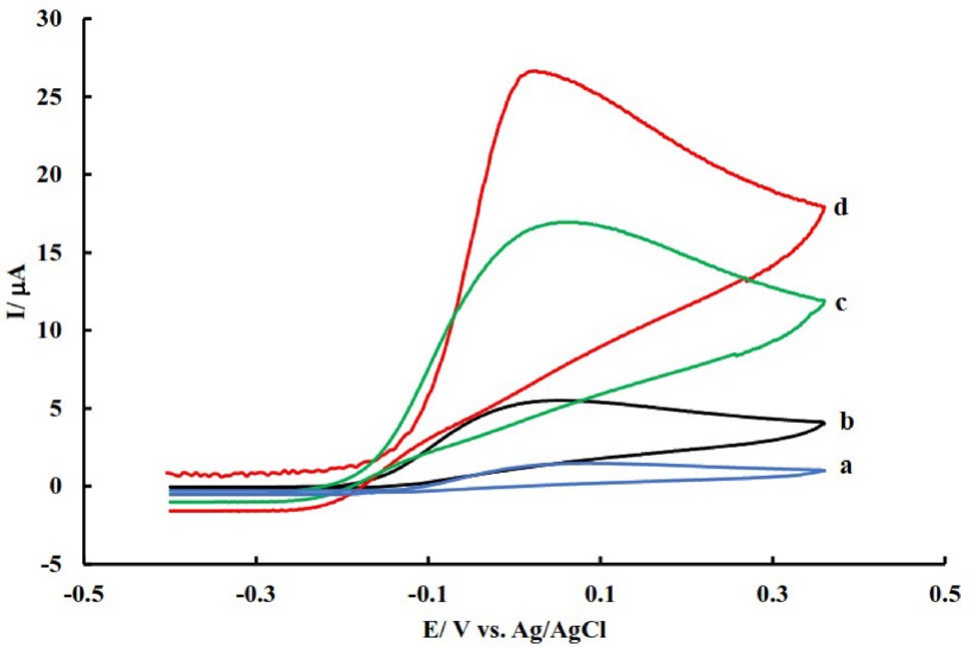
Cyclic voltammograms of unmodified GCE (**a**), N-GQD/GCE (**b**), PdNPs@GQD/GCE (**c**), and PdNPs@N-GQD/GCE (**d**) in PBS containing 45 μM AA.

To investigate the effect of different modifiers in the electrocatalysis of AA, the electrochemical behavior of different electrodes was investigated in 45 μM of AA solution. As shown in the resulting voltammograms (Fig. 5), there is a small peak for AA oxidation on the bare electrode at a potential approximately equal to 0.1 V. After modification of the electrode with N-GQD, the AA oxidation current was increased may be due to the higher surface area and electron transfer rate of doped nanomaterials compared to unmodified electrode. After the immobilization of PdNPs decorated quantum dots, AA oxidation potential shifts to less positive values due to the catalytic effect of PdNPs as a modifier and fast electron transfer rate of the modifier. Also, the results show that the GCE modified with PdNPs@N-GQD shows better electrocatalytic activity for the oxidation of AA and the oxidation current of AA at the surface of this electrode was significantly higher than PdNPs@GQD/GCE (Fig. 5). The better signal obtained on the surface of PdNPs@N-GQD/GCE compared to PdNPs@GQD/GCE confirms that nitrogen increases the charge carriers and the interaction between the support and metal nanoparticles and the withdrawal of electrons necessary to increase the activity of metal nanoparticles. Generally, these result in the higher electrocatalytic activity of the proposed sensor.

The electrochemical response of the PdNPs@N-GQD/GCE after addition different concentrations of AA was studied. Figure S2-a shows the obtained CV after the addition of different concentrations of AA at the surface of the Pd@N-GQD/GCE and the resulting calibration curve Fig. S2-b proves linear dependency of signal to AA concentration in the range from 5 to 45 μM (R^2^ = 0.99).

**Figure 6:**
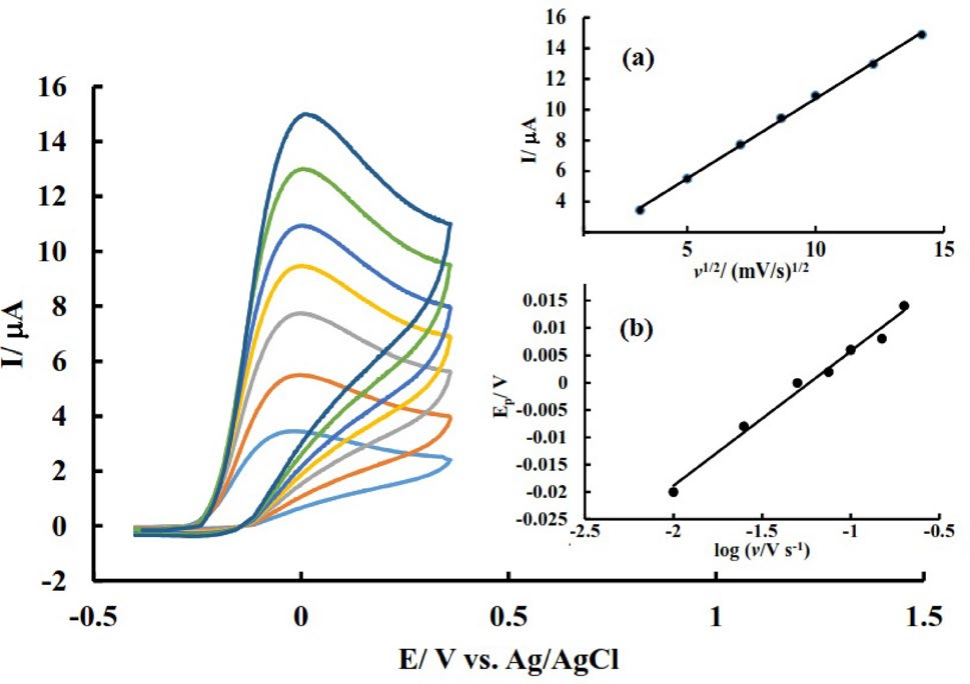
CV of PdNPs@N-GQD/GCE in the 12 μM of AA (PBS, pH 7) using different scan rates; Inset a: variation of signals versus square root of scan rate, Inset b: variation of oxidation peak potential versus logarithm of the scan rate.

It is worth mentioning that in a diffusion-controlled process, the current of the electroactive species is proportional to the second root of the potential sweep rate, while for a surface-adsorbed species, the current changes proportionally to the potential sweep rate. Figure 6a shows obtained voltammograms using different scan rates and the variation of signals versus the square root of the scan rate.

As seen in Fig. 6a, the current of the AA oxidation signal changes linearly with the second root of the potential sweep rate (R^2^ = 0.99), so it can be understood that the AA oxidation process on the surface of this electrode is diffusion controlled. On the other hand, since the potential of the oxidation peaks of AA is slightly shifted towards more positive potentials in proportion to the potential scanning speed, it can be understood that the electrochemical oxidation of AA is limited by the charge transfer kinetics between the electrode and the electroactive species. For a completely irreversible reaction, the relationship between the speed of the potential scan and the peak potential is determined with the help of the following equation:

$${\text{E}}_{{\text{p}}} = \left( {{\text{b}}/{2}} \right){\text{ log}}\;v + {\text{Const}}.$$

where E_p_ is the peak potential, b is the TOEFL slope, and *v* is the speed of the potential scan. Figure 6b shows changes in the anodic peak potential of AA versus to logarithm of the potential sweep rate. The value of the obtained slope for this line is equal to 0.025 V/decade, therefore the Tafel slope will be equal to:

$${\text{b}} = 0.0{25} \times {2} = 0.0{5 }\left( {{\text{V}}/{\text{decade}}} \right)$$

**Figure 7:**
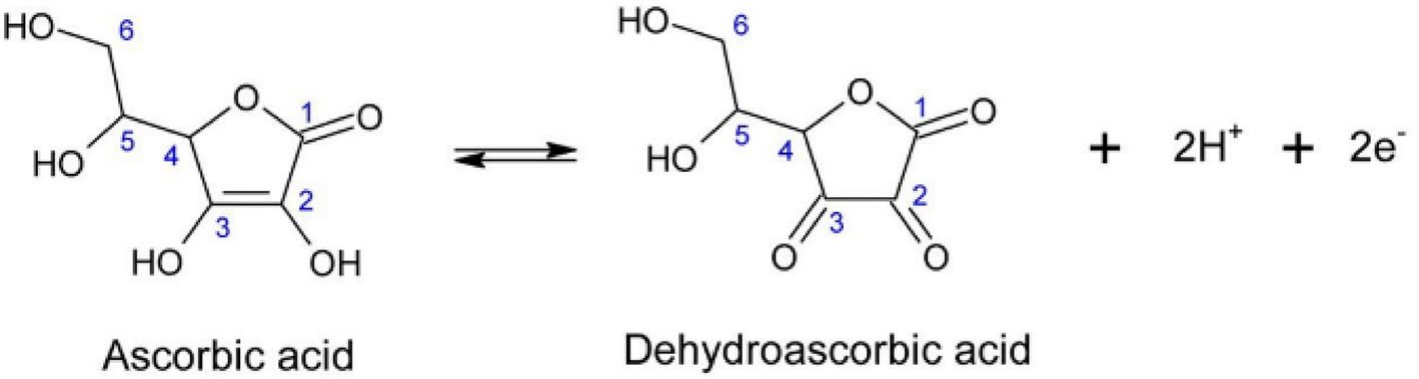
The proposed oxidation mechanism of AA.

By placing the Tafel slope in the expression b = 2.3RT/n_α_F(1 − α) and assuming 2 for the number of electrons involved in the rate-determining step (n_α_), the charge transfer coefficient, α, calculated equal to 0.41. Based on these results, the following mechanism was proposed for the oxidation of AA at the surface of the proposed electrode (Fig. 7):

### Investigating the effect of pH on AA oxidation

**Figure 8:**
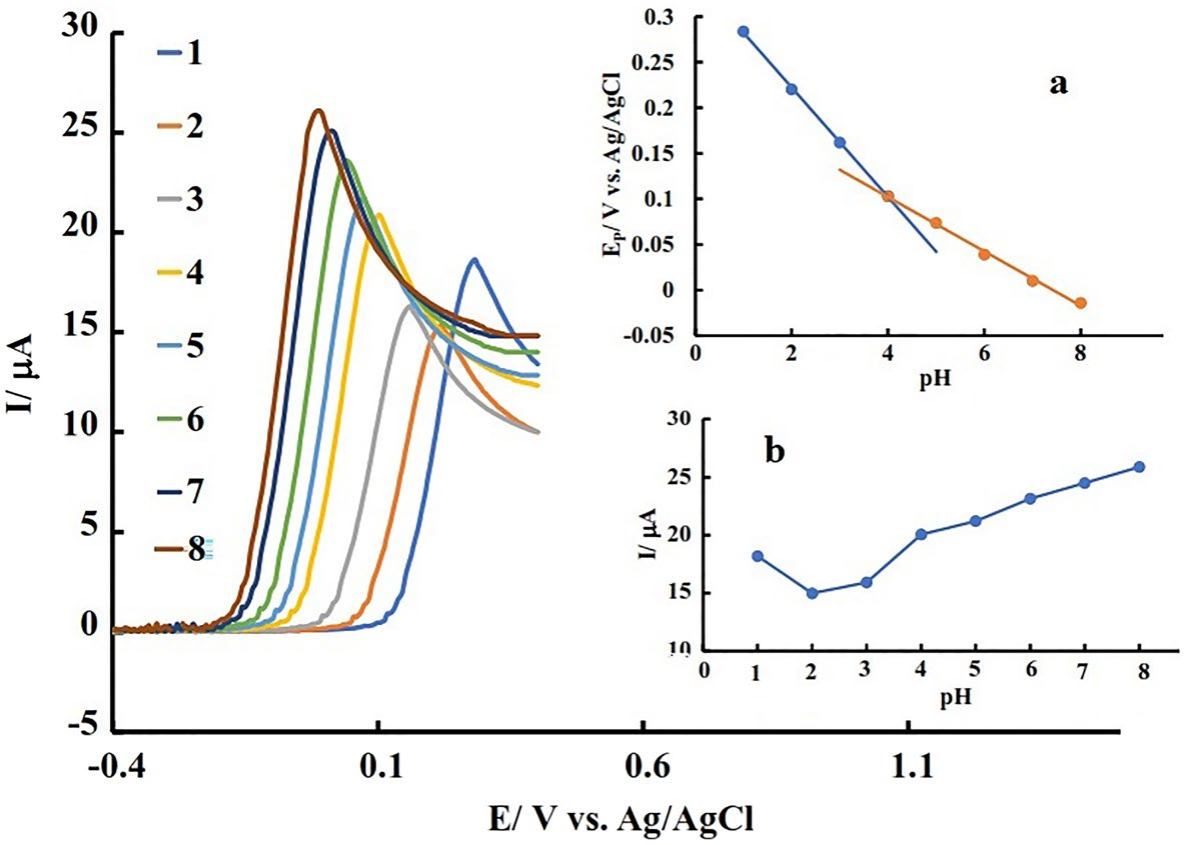
CV of PdNPs@N-GQD/GCE in the AA containing PBS (45 μM) with different pH values; inset a: variation of oxidation peak potential (E_P_) and b: variation of oxidation peak current versus applied pH values.

In order to investigate the effect of pH on the oxidation of AA, solutions with concentrations of 0.6 μM were prepared in the background electrolyte, and their pH was adjusted to different values by a solution of sodium hydroxide (NaOH) or hydrochloric acid. The linear sweep voltammograms of the PdNPs@ N-GQD/GCE were recorded inside these solutions. The resulting voltammograms are shown in Fig. 8 and the graph of changes in E_pa_ of the voltammograms according to pH is shown in Fig. 8a. Based on this diagram, the changes of E_pa_ versus pH consisted of two linear regions in the range of 4 > pH > 1.

In the pH ≤ 4, the slope of E_Pa_ changes in terms of pH is equal to 60 millivolts, which indicates a two-electron, two-proton reaction. In the range of 4 < pH < 8, the slope of E_pa_ versus pH is equal to 29 mV, which indicates a two-electron, one-proton reaction. According to the proposed oxidation mechanism for AA, in the alkaline medium, the presence of OH^−^ due to the interaction with the produced protons, shifts the equilibrium towards the product which leads to the chang of oxidation potential towards low positive values and an increase in the obtained signal. It should be mentioned that higher pH values due to the natural pH of body fluids have not been examined and pH 7 was selected as the optimum value for AA measurement (Fig. 8b).

### Analytical application of the sensor

For measuring the low concentrations of AA and achieving low LOD values, differential pulse voltammetry (DPV) was used. For this purpose, the DPV results of the proposed modified electrode in the presence and absence of AA are shown in Fig. S3. As presented, an AA oxidation signal appeared with the AA added to the blank electrolyte solution.

To ensure the absence of analyte adsorption on the electrode surface, the possibility of analyte adsorption was also investigated applying different times and potentials for pre-concentration. In the adsorption reactions, by varying the adsorption time or potential the number of adsorbed species varies. Consequently, the effect of these parameters was studied by placing prepared electrodes in the 5 μM AA solution and applying different pre-concentration potentials or pre-concentration times. Results (Figs. S4 and S5) showed that increasing the pre-concentration time and variation of preconcentration potential have no effect on the AA oxidation signal at the electrode surface which indicates the absence of AA absorption at the electrode surface.

### Investigating the effect of analyte concentration

**Figure 9:**
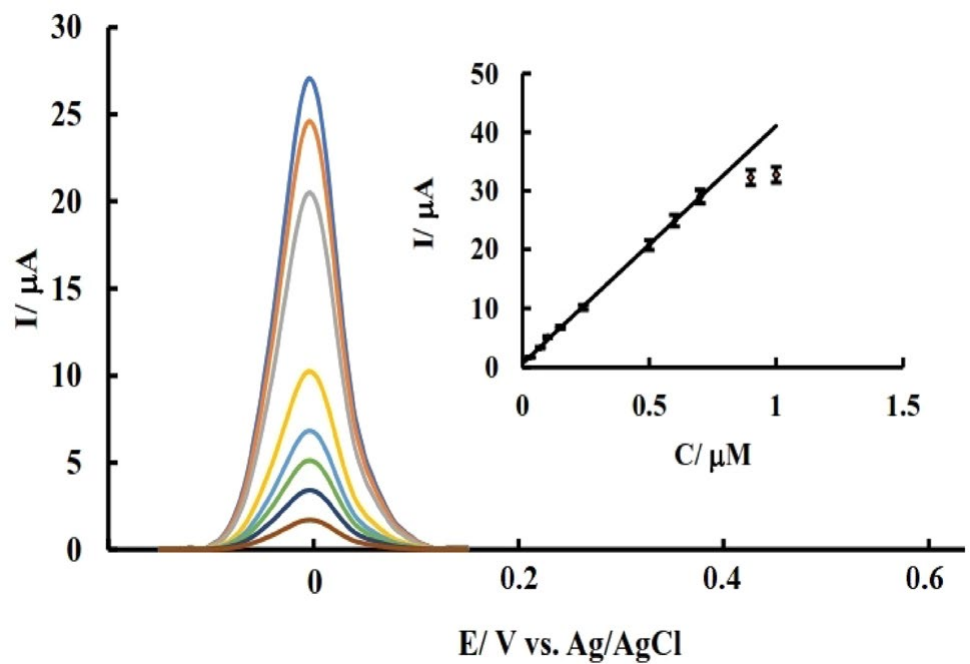
DPV results of PdNPs@N-GQD/GCE in the PBS after the addition of various concentrations of AA.

The use of each electrode and obtaining experimental data is a topic that is closely related to the ability to use that electrode in real environments. Real environments contain their analytes, and each sensor demonstrates its high reliability by being able to detect small amounts of analytes in the presence of other compounds. DPV results for PdNPs@N-GQD/GCE in the presence of adding amounts of AA are shown in Fig. 9. As can be seen in the graphs, the resulting current was elevated with increasing concentration from 0.03 μM to 1 μM and this relationship was linear in the concentration range from 0.03 to 0.7 μM (R^2^ = 0.99). Based on the calibration curve slope (sensitivity) and RSD of the obtained calibration curve (signal versus the concentration), the detection limit of the sensor was calculated as 23 nM which is comparable with other works abstracted in Table S1. As can be seen in Table S1, besides simple preparation, good stability, and selectivity (explained in continue), the LOD value of this sensor is comparable to other sensors proposed for AA determination.

### Interference check

**Figure 10:**
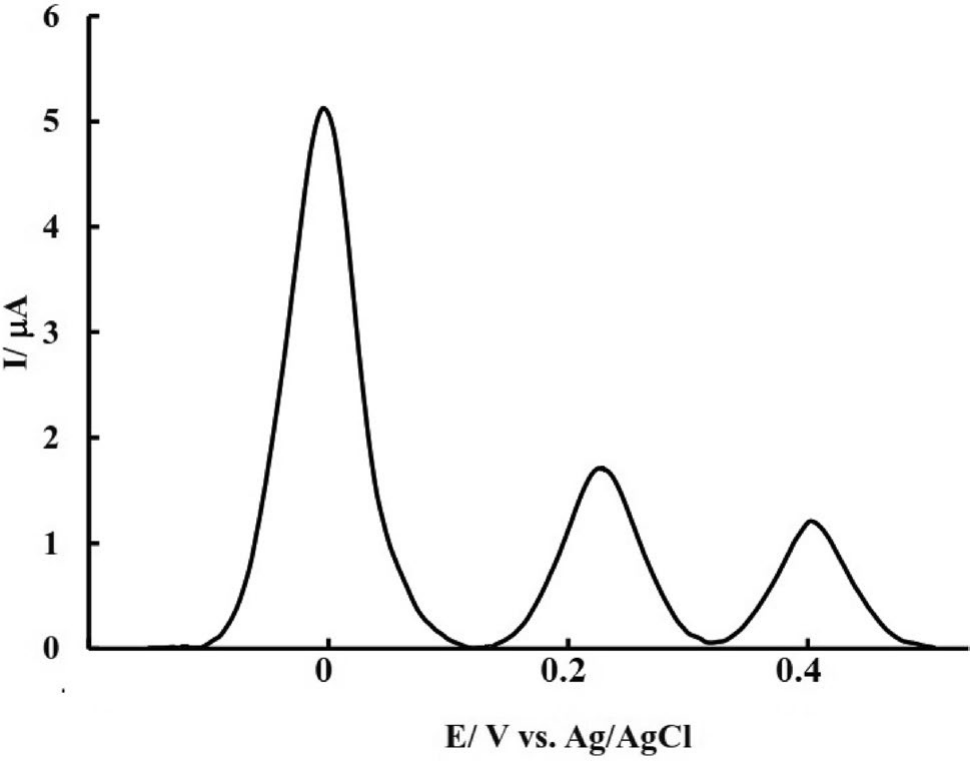
DPV of PdNPs@N-GQD/GCE in the PBS after addition after adding 0.1 μM of AA, 2 μM of UA and 2 μM of DA.

In order to investigate the effect of interference, the DPV of 10 ml background electrolyte was recorded twice in the presence of AA, once in the absence, and again in the presence of possible interfering species, and the obtained signals were compared. If the signal of the analyte was changed less than 5% in the presence of interfering agents, it can be concluded that the examined species do not affect the measurement of the analyte. The DPV results of evaluated probable interfering compounds such as UA and dopamine (DA) which are present in biological fluids with AA were presented in Fig. 10 and results prove different sensitivity and oxidation potential for DA and UA which result in high selectivity of the proposed sensor. In addition, according to the results (Fig. S6), AA could be measured selectively using the proposed sensor in the presence of other probable interfering species such as citric acid (CA), oxalic acid (OA), DA, UA, Fe(II) and a mixture of them.

### Application of modified electrode in measuring AA in real samples

The application of the modified electrode as a voltammetric sensor in the measurement of AA has been evaluated in some fruit juice and some pharmaceutical samples. To measure AA in a chewable tablet sample, the first three tablets were carefully weighed, and the average weight of the tablets was determined. Then these tablets were powdered and mixed in a mortar until they became completely uniform. Then 0.075 g of powder was weighed and made up to volume in a 25 ml flask with distilled water. Then it is successively diluted with the electrolyte solution until the concentration is in the linear range of the calibration curve. A specific volume of this solution was added to the electrochemical cell and after stirring, the voltammetric signal was recorded. Afterward, increasing amounts of the standard AA solution were added to the cell, and next to each volume addition and suitable stirring, the signal was recorded again. Analyte concentration was determined by drawing a signal change diagram according to the concentration. Applying the x-intercept of the resulting curve, and considering the amount of sample and preparation steps, the milligrams of AA in each tablet were calculated. To ensure the accuracy of the results, the samples were spiked with a standard AA solution and the analyte in the spiked solutions was measured using the standard addition method, the results of which are summarized in Table 1. Based on the obtained results the amount of AA in 100 ml orange juice and one chewing tablet was calculated at about 53 mg and 249.8 mg.

**Table 1:** Results for the determination of AA in chewing tablet and fruit juice samples.

Sample	Found (nM)	Added (nM)	Recovery (%)	RSD (%) [a]
Chewing tablet (250 mg/tablet)	40	0		3.12
	44.98	5	99.9	3.65
	50.34	10	101.13	3.03
Orange juice	53.32 mg/100 ml	0		2.93
	62.91	10	99.35	3.21
	73.01	20	99.57	2.46

### Checking the stability of the electrode

The repeatability of PdNPs@N-GQD/GCE was checked by the repetition of the voltammetric test five times in a 0.1 μM solution in the optimum conditions, and the obtained relative standard deviation (RSD) equal to 3.15%, established the repeatability of the proposed sensor. The prepared electrode can be reused after simple washing with deionized water and does not require extra treatment. Due to the good repeatability of the prepared sensor, it is possible to use it 19 times of measurement (Fig. S7).

The reproducibility of the sensor was evaluated by considering the RSD of the results obtained from five freshly prepared PdNPs-N-GQD/GCE (Fig. S8), and a value equal to 4.05% indicated the good reproducibility of the proposed sensor.

The stability of PdNPs@N-GQD/GCE was evaluated by keeping the prepared electrode at 4 °C and using it for measurement on different days (first, second, etc.) after preparation (Fig. S9). Obtaining 95% of the initial response after 5 days is a confirmation of the stability of the sensor up to 5 days. Considering the possibility of reusing this sensor without the need for treatment steps, 5 days was considered a satisfactory time for the current sensor.

## Conclusions

In this study, nitrogen-doped nanoparticles were synthesized from the condensation reaction of citric acid in the presence of ethylenediamine. The prepared N-doped GQD was subsequently modified by palladium nanoparticles. Various microscopic methods were used to distinguish the synthesized nanoparticles and a novel electrochemical sensor was prepared using these nanoparticles presented as PdNPs@N-GQD/GCE. The electrochemical behavior of AA and its sensitive determination was studied using this prepared sensor. Considering the synergistic effect of Pd nanoparticles as an active electrocatalyst, and N-GQD as an electron transfer accelerator and electrocatalytic activity improving agent, PdNPs@N-GQD hybrid materials showed excellent activity in the direct oxidation of AA.

Under optimal conditions, the LOD of the proposed sensor was 23 nm and used to determine the concentration of AA in real samples of chewable tablets and fruit juice.

The results showed that in optimal conditions, the proposed sensor can be used for simultaneous and selective determination of AA, UA and DA.

### Supplementary Information

Supplementary Information.

## Data Availability

All data generated or analyzed during this study are included in this published article and its supplementary information files.
