# Supplementary material for: Electrochemical determination of ascorbic acid using palladium supported on N-doped graphene quantum dot modified electrode
Source: Sci Rep. 2024 Mar 12;14:5982. doi: 10.1038/s41598-024-56231-x (PMC10933321; doi:10.1038/s41598-024-56231-x)
Supplement: Supplementary file 1 — Supplementary Information. [file 41598_2024_56231_MOESM1_ESM.docx]

**Electrochemical determination of ascorbic acid using palladium supported on N-doped graphene quantum dot modified Electrode**

K. Mohammadnezhad^a,b^, F. Ahour^a,b^*, S.Keshipour^a,b,c^

^a^Nanotechnology Research Group, Faculty of Chemistry, Urmia University, Urmia, Iran

^b^Department of Nanochemistry, Nanotechnology Research Center, Urmia University, Urmia, Iran

^c^Central Laboratory of Urmia University, Urmia University, Urmia, Iran.

*Email: F.Ahour@urmia.ac.ir; Fatemeh.ahour@gmail.com

Corresponding author:

* Fatemeh Ahour

E-mail: [Fatemeh.ahour@gmail.com](mailto:Fatemeh.ahour@gmail.com), f.ahour@urmia.ac.ir; Fax: +98 44-32752746

**Experimental**

Instrumentation and reagents

To perform electrochemical tests, AUTOLAB PGSTAT 30 electrochemical analysis system was used, which is connected to the controlling computer through GPES 4.9 software package. A three-electrode set consisting of PdNPs@N-GQD modified glassy carbon electrode (diameter 3 mm) as working electrode, Ag/AgCl (1 M KCl) and platinum wire as a reference, and auxiliary electrodes respectively were used in experiments.

A Nicolet FT-IR NEXUS 670 spectrometer (Thermo Scientific, USA) was used to record IR spectra and identify different functional groups in the synthesized compounds. MIRA III scanning electron microscope connected to EDX-Line scan map was used to perform field effect scanning electron microscope (FE-SEM) and energy dispersive X-ray spectroscopy (EDX). The pH value was measured using a digital pH meter (HANNA 212). The GCE surface was cleaned using an ultrasonic bath (KODO model JAC1002). This device was also used to prepare the homogenous solution of the modifier. All chemicals with analytical grade were obtained from Merck, Germany. Deionized water was used to prepare all solutions. AA solutions were prepared freshly just before experiments. PBS (0.5 M phosphate buffer) was prepared using Na_2_HPO_4_ and NaH_2_PO_4_ salts at the beginning of laboratory work.

Electrode modification

Firstly, rubbing with a polishing cloth used for cleaning the surface of the working electrode. In continue, polished electrode sonicated for 5 minutes and carefully washed with doubly distilled water. To prepare the modifier suspension, 10 mg of PdNPs@N-GQD was added was into 10 mL deionized water (pH 4) and sonicated for 10 min till to disperse the PdNPs@N-GQD completely. The modified electrode was organized according to the procedure reported in the previous work.

In this way, the GCE was placed inside the homogenous solution of Pd@N-GQD (1 mg ml^-1^) and 70 potential cycles were performed in the range of 0 to 1 at a speed of 100 mV s^-1^. The electrodeposition of PdNPs@N-GQD on the electrode surface can be related to the electrostatic interactions between the surface and negatively charged groups of PdNPs@N-GQD. The modified electrode was washed with doubly distilled water and kept at the refrigerator before use.

Voltammetric measurements

For CV (cyclic voltammetry) measurements, AA was added in electrolyte containing electrochemical cell and analysed using potential scanning in the range from -0.4 to 0.35 V. In DPV analysis, potential scanned from -0.4 to 0.4 V with the best DPV parameters as 0.4 s interval time, 0.05 s modulation time, 25 mV potential amplitude, and 5 mV step potential. All the experiments were done at least 3 times and the presented results are the average of these replicates with the corresponding error bars in the figures.


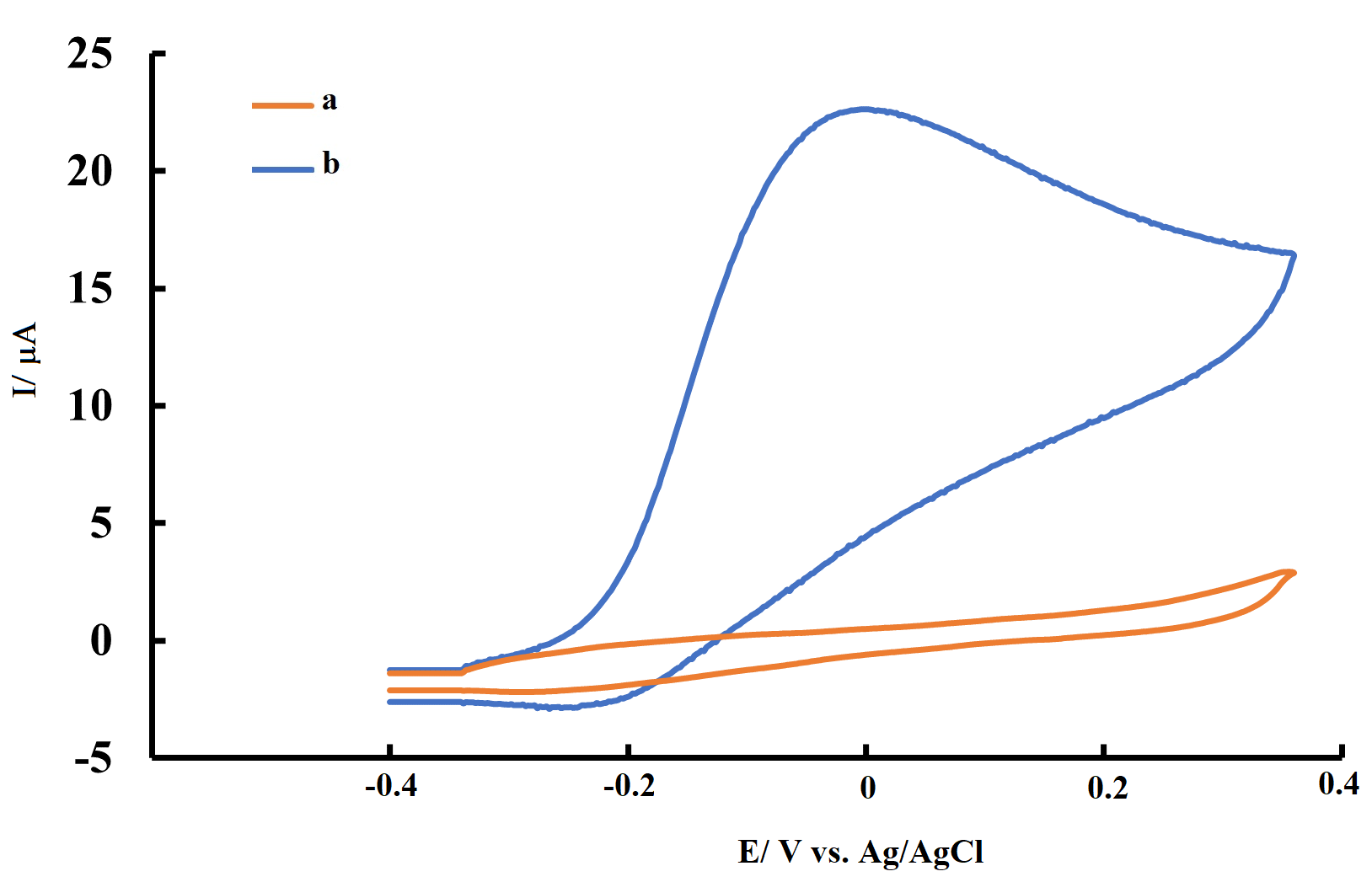


**Fig. S1.** Cyclic voltammograms of the [PdNPs@N-GQD/GCE in 0.5](mailto:PdNPs@N-GQD/GCE%20in%20.4) M PBS before and after addition of 0.04 mM AA; Scan rate: 100 mV s^-1^.


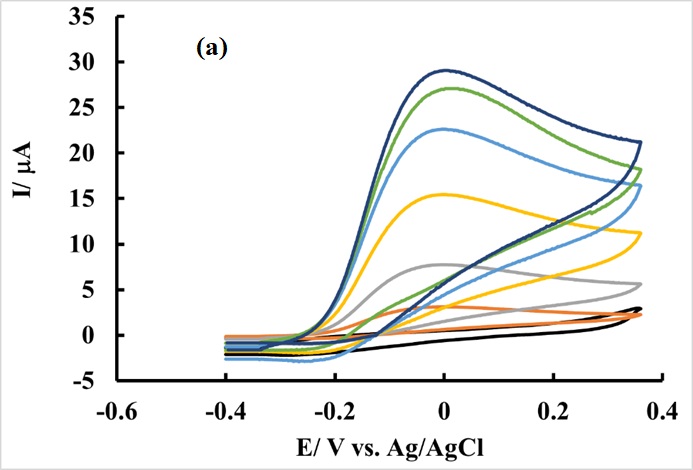


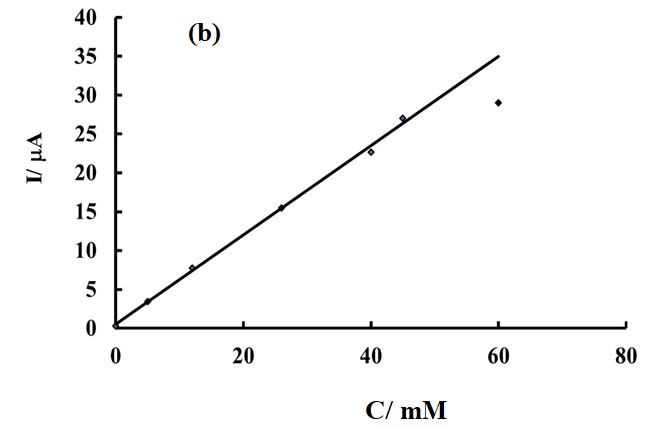


**Fig S2**. Cyclic voltammograms of PdNPs@N-GQD/GCE after addition different concentrations of ascorbic acid in the range from 5 to 45 μM (a) and resulting calibration curve (b)


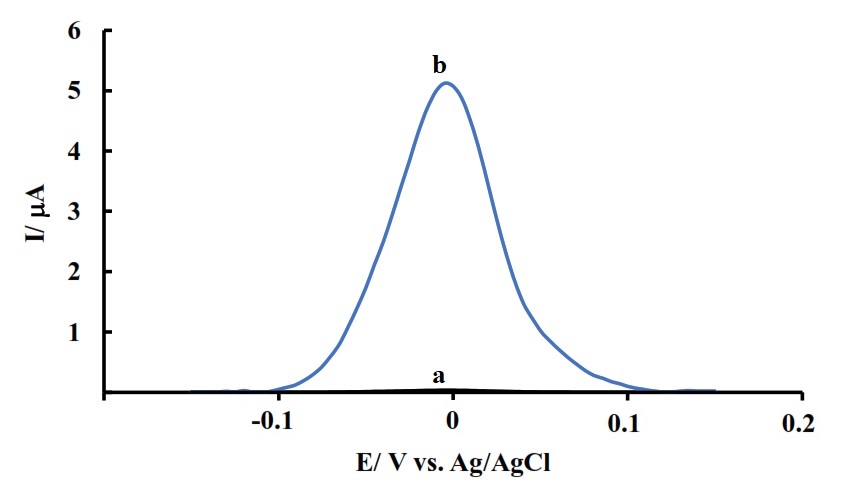


Figure S3. DPV results of the PdNPs@N-GQD/GCE obtained in background electrolyte in the (a) absence and (b) presence of 0.1 μM of ascorbic acid.


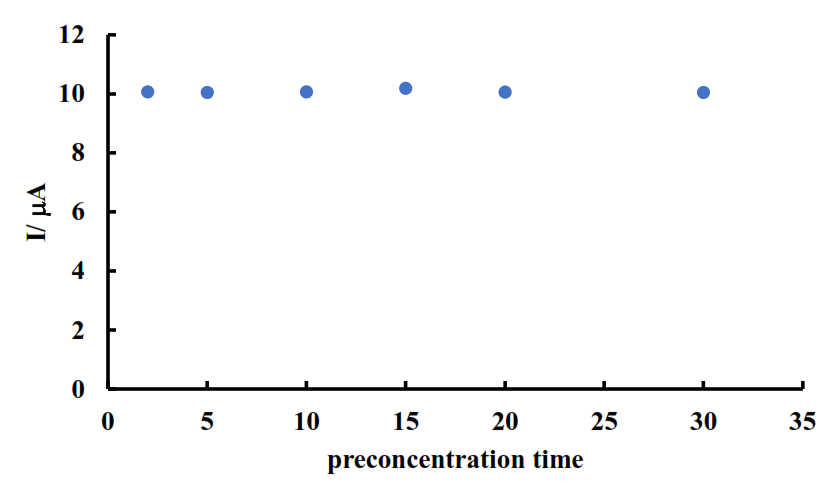


Fig. S4. Variation of ascorbic acid oxidation signal (0.24 μM) at the surface of PdNPs@N-GQD/GCE versus preconcentration time. PBS with pH 7 as electrolyte and applying open circuite condition.


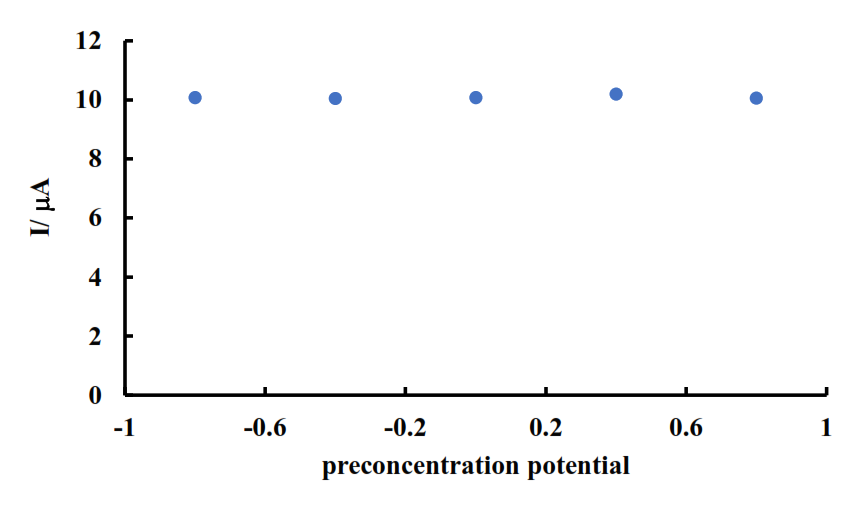


Fig. S5. Variation of DPV oxidation signal of 0.24 μM AA at the surface of PdNPs@N-GQD/GCE versus preconcentration potential. PBS with pH 7 as electrolyte and using 5 min as preconcentration time.

**Table S1**. Analytical parameters of PdNPs@N-GQD/GCE compared to other modified electrodes

| Modifier | Linear range | LOD | Ref. |
| --- | --- | --- | --- |
| Pd/Al* | 1-50 µM | 0.5 µM | 1 |
| CeO_2_-MWCN | 0.005-0.539 µM | 8 nM | 2 |
| CdO-SPCE* | 5 to 150 µM | 53.5 nM | 3 |
| PdNW* | 25 mM–0.9 mM | 0.2 mM | 4 |
| PdNPs-GO* | 0.02 to 2.28 mM | ͟ | 5 |
| Pd/CNFs* | 0.05–4 mM | 15 μM | 6 |
| PdNPs@N-GQD | 0.03-0.7 μM | 23 nM | This work |

Pd/Al: palladized Al electrode; CdO-SPCE: cadmium oxide nanoparticles decorated screen-printed carbon electrode; PdNW: Pd nanowire; PdNPs-GO: palladium nanoparticles supported on graphene oxide; Pd/CNFs: palladium nanoparticle-loaded carbon nanofibers


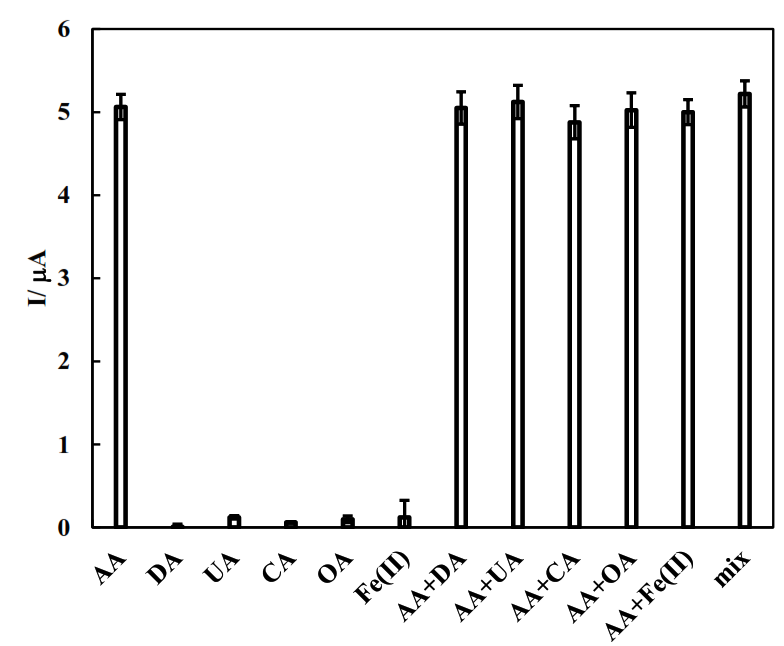


**Fig. S6**. Histogram related to DPV response of PdNPs@N-GQD/GCE dipped in PBS with pH 7 after addition of 0.1 μM AA and 1 μM of other possible interfering compounds and mixtures thereof as mentioned.


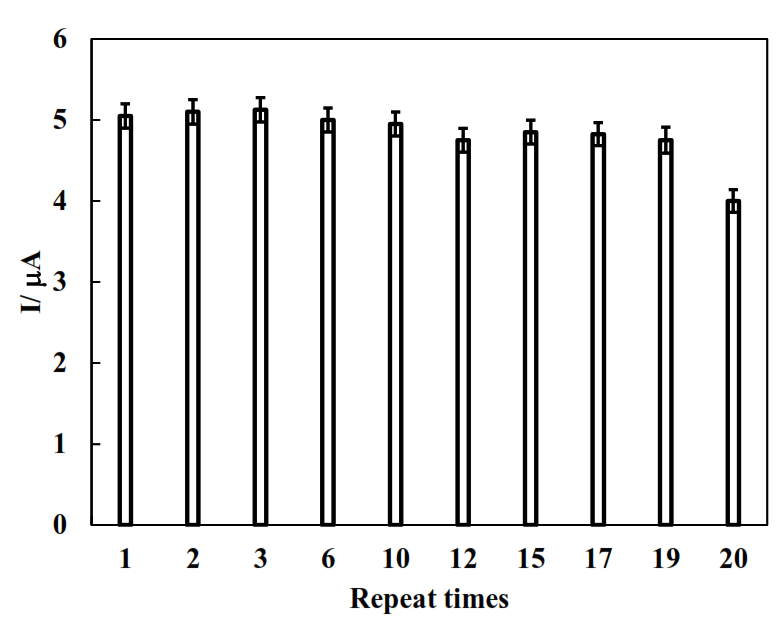


**Fig. S7.** Histogram of DPV signal of PdNPs@N-GQD/GCE after immersion in 0.1 μM AA and signal recording in different repetitions. Condition: PBS pH 7.


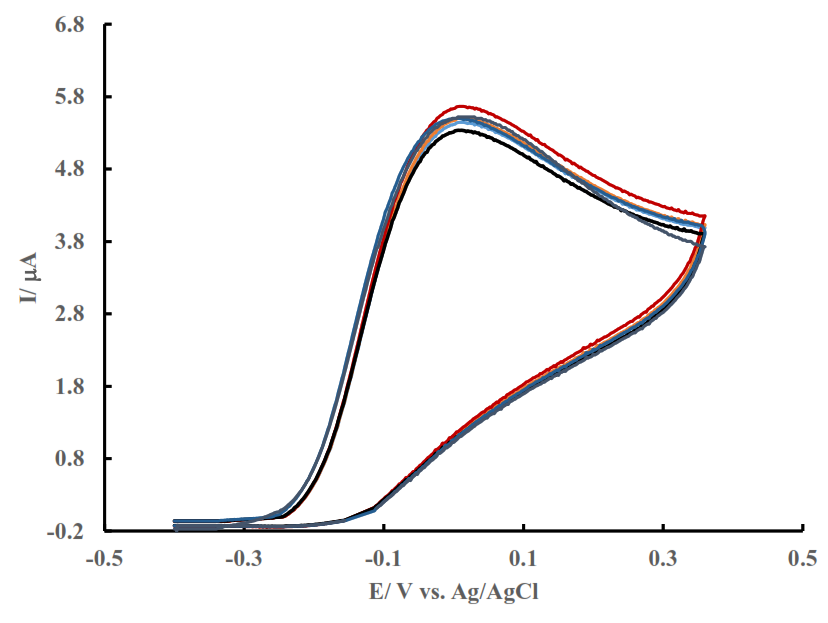


**Fig. S8.** Cyclic voltammograms of the five newly prepared [PdNPs@N-GQD/GCE after immersion in 0.5 M PBS containing 8 μM of AA](mailto:PdNPs@N-GQD/GCE%20in%20.4); Scan rate: 100 mV s^-1^.


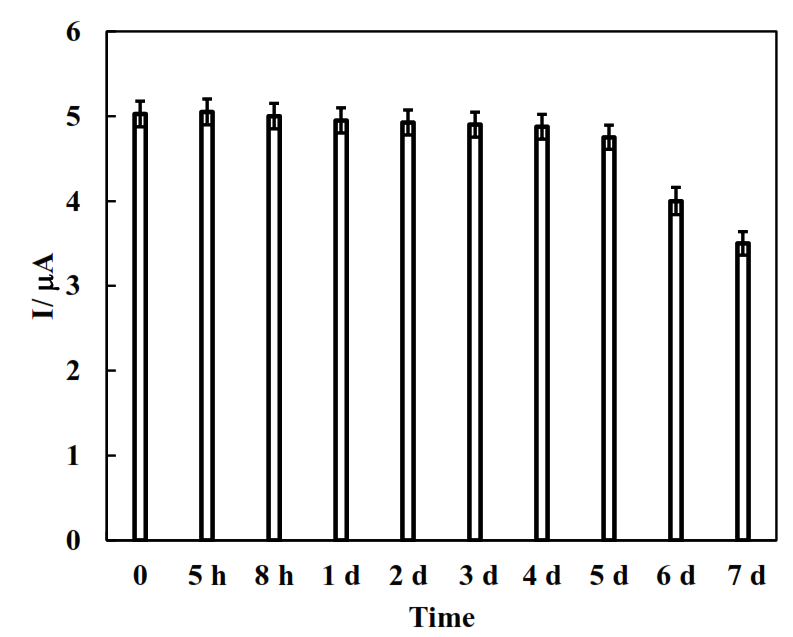


**Fig. S9.** Histogram related to DPV signal of *of* PdNPs@N-GQD/GCE after immersion in 0.1 μM of AA using newly prepared electrode and after storage for different time intervals. Conditiong: PBS pH 7.

**REFERENCES**

1. Pournaghi‐Azar, M.H., Dastangoo, H., Fadakar, R. Differentiation of detection of ascorbic acid and dehydroascorbic acid using hydrodynamic amperometry and anodic stripping voltammetry on modified aluminum electrodes. *Electroanalysis* ***22***, 229-235, DOI: <https://doi.org/10.1002/elan.200900213> (2010).
2. Sangsefidi, F.S., Salavati-Niasari, M., Mazaheri, S., Sabet, M. Controlled green synthesis and characterization of CeO_2_ nanostructures as materials for the determination of ascorbic acid. *J. Mol. Liq.* **241**, 772-781, DOI: <https://doi.org/10.1016/j.molliq.2017.06.078> (2017).
3. Gopalakrishnan, A., Sha, R., Vishnu, N., Kumar, R., Badhulika, S. Disposable, efficient and highly selective electrochemical sensor based on cadmium oxide nanoparticles decorated screen-printed carbon electrode for ascorbic acid determination in fruit juices. Nano-Struct. Nano-Objects **16**, 96-103, DOI: <https://doi.org/10.1016/j.nanoso.2018.05.004> (2018).
4. Wen, D., Guo, S., Dong, S., Wang, E. Ultrathin Pd nanowire as a highly active electrode material for sensitive and selective detection of ascorbic acid. *Biosens. Bioelectron.* **26**, 1056-1061, DOI: <https://doi.org/10.1016/j.bios.2010.08.054> (2010).
5. Wu, G.H., Wu, Y.F., Liu, X.W., Rong, M.C., Chen, X. M., Chen, X. An electrochemical ascorbic acid sensor based on palladium nanoparticles supported on graphene oxide. *Anal. Chim. acta* **745**, 33-37, DOI: <https://doi.org/10.1016/j.aca.2012.07.034> (2012).
6. Huang, J., Liu, Y., Hou, H., You, T. Simultaneous electrochemical determination of dopamine, uric acid and ascorbic acid using palladium nanoparticle-loaded carbon nanofibers modified electrode. *Biosens. Bioelectron.* **24**, 632-637, DOI: <https://doi.org/10.1016/j.bios.2008.06.011> (2008).
